# Third harmonic generation imaging and analysis of the effect of low gravity on the lacuno-canalicular network of mouse bone

**DOI:** 10.1371/journal.pone.0209079

**Published:** 2019-01-02

**Authors:** Rachel Genthial, Maude Gerbaix, Delphine Farlay, Laurence Vico, Emmanuel Beaurepaire, Delphine Débarre, Aurélien Gourrier

**Affiliations:** 1 Univ. Grenoble Alpes, CNRS, LIPhy, Grenoble, France; 2 INSERM U1059, Université de Lyon, St Etienne, France; 3 French National Centre for Space Studies, Paris, France; 4 INSERM, UMR1033, Université de Lyon, Lyon, France; 5 Lab. for Optics and Biosciences, Ecole Polytechnique, CNRS, INSERM, Palaiseau, France; Pennsylvania State Hershey College of Medicine, UNITED STATES

## Abstract

The lacuno-canalicular network (LCN) hosting the osteocytes in bone tissue represents a biological signature of the mechanotransduction activity in response to external biomechanical loading. Using third-harmonic generation (THG) microscopy with sub-micrometer resolution, we investigate the impact of microgravity on the 3D LCN structure in mice following space flight. A specific analytical procedure to extract the LCN characteristics from THG images is described for ex vivo studies of bone sections. The analysis conducted in different anatomical quadrants of femoral cortical bone didn’t reveal any statistical differences between the control, habitat control and flight groups, suggesting that the LCN connectivity is not affected by one month space flight. However, significant variations are systematically observed within each sample. We show that our current lack of understanding of the extent of the LCN heterogeneity at the organ level hinders the interpretation of such investigations based on a limited number of samples and we discuss the implications for future biomedical studies.

## Introduction

Bones are complex biological structures able to simultaneously achieve diverse functions including mechanical support, calcium storage and red blood cell production in the marrow. They are structured following a nanocomposite, multiscale, hierarchical pattern [1] which results from the intricate cellular mechanisms underlying bone growth. During tissue formation or regeneration, a subpopulation of osteoblasts (bone forming cells) become embedded in the collagenous extracellular matrix and differentiate into osteocytes that exhibit a dendritic phenotype, similar to neurons. Mature bone tissue thus hosts a dense network of interconnected osteocytes which have been shown to play a key role in mechanosensing of macroscopic strain and calcium mineral regulation [2].

**Fig 1:**
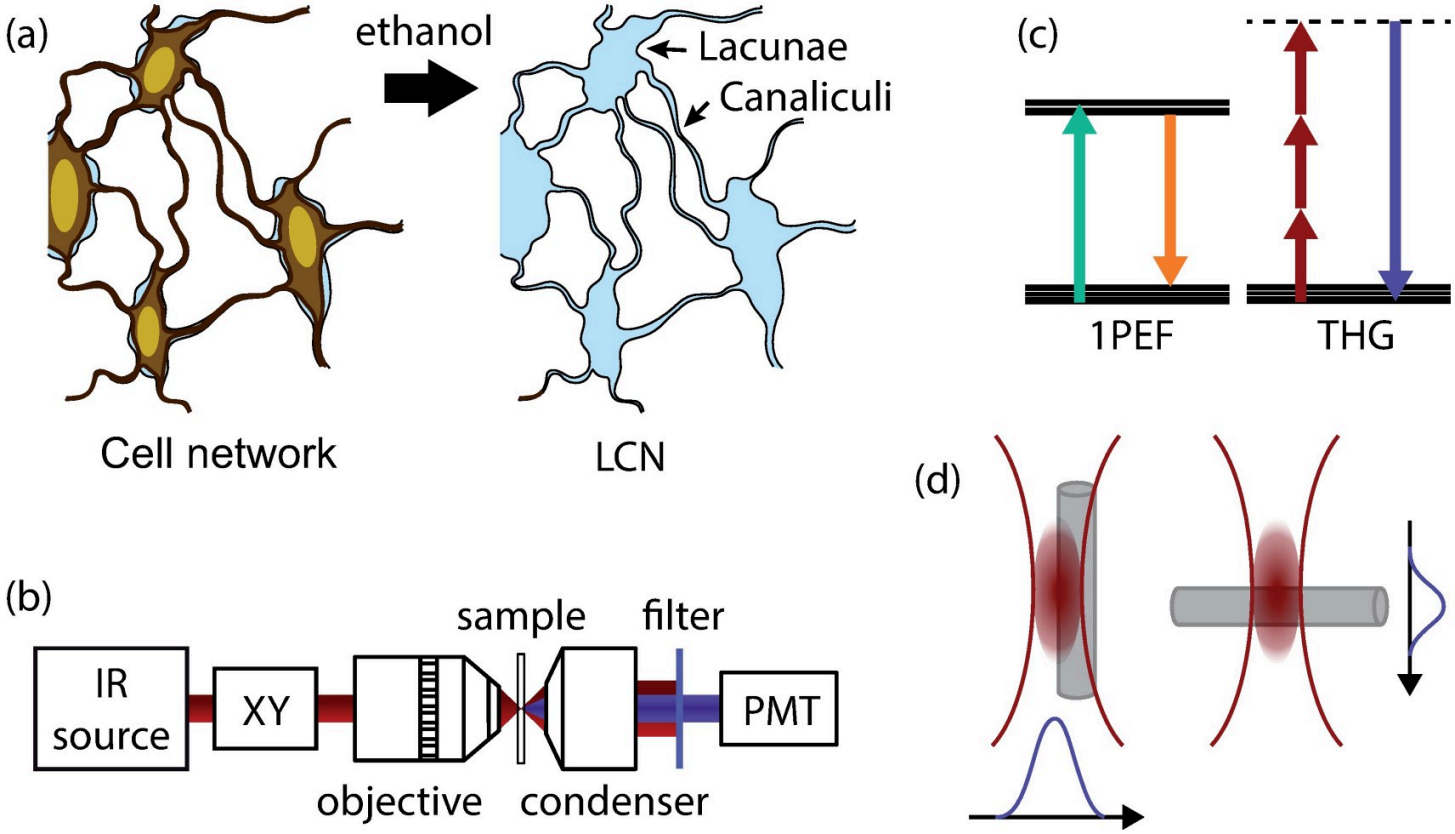
Bone structure, THG microscopy setup and principle. (a) simplified view of the osteocyte network in cortical bone (left) and the corresponding cavities in the bone matrix forming the lacuno-canalicular network (LCN). (b) Microscopy setup: a pulsed infrared 1180 nm beam is scanned and focussed on the sample, and the THG signal is collected in transmission onto a photomultiplier tube (PMT) using a 1.4NA condenser. (c) simplified Jablonski diagrams for single-photon excited fluorescence used in our confocal microscopy experiments (1PEF, left), and third-harmonic generation (THG, right). The THG signal is created at exactly one third of the excitation wavelength. Dotted line, virtual energy level. (d) the coherent buildup of the THG signal and the excitation phase shift of the excitation beam at the focal spot result in a non-zero signal only in the presence of optical heterogeneities within the focal volume, as created by canaliculi in the mineralized collagen bone matrix. This signal is strongly dependent on the canaliculi diameter and orientation.

The structure of this osteocyte network recently received a renewed attention due to new possibilities offered by sub-micron spatial resolution imaging using X-rays, electrons or optical probes [3]. In most cases, the analysis is based on the visualization of the porosity arising from the cell network (Fig 1): the lacunae surrounding the osteocyte bodies and the canaliculi hosting the processes connecting the osteocytes forming the lacuno-canicular network (LCN). The challenge in studying this network essentially lies in the necessity to achieve a sub-micrometric spatial resolution to resolve the canaliculi in 3D. Furthermore, fields of view compatible with histology (up to ∼ 1 cm^2^) must be achieved, in order to relate the cellular network with the heterogeneous tissue structure. To this aim, confocal fluorescence microscopy can be used to visualize a fluorescent dye which has penetrated the LCN [4–6]. However, such methods fail when the sample is embedded in resin for sectioning purposes prior to histological analysis, since the resin generally infiltrates the LCN. Therefore, large collections of embedded bone samples available in biomedical laboratories cannot be used *a posteriori* for LCN analysis.

Due to their intrinsic optical sectioning and greater penetration depth compared to confocal microscopy, non-linear optical imaging methods such as two-photon fluorescence (2PEF) and second-harmonic generation (SHG) microscopies are increasingly used in bone studies. Over the past two decades, THG microscopy has emerged as a powerful technique to characterize interfaces in biology without the need for staining. The signal creation relies on the nonlinear scattering of three incident (usually infrared) photons that creates a (usually visible) photon in the presence of optical heterogeneities at the scale of the excitation focal volume (Fig 1d). THG application areas in biological imaging range from embryogenesis [7–9] to cell migration [10], with a large number of studies on its possible use for virtual optical biopsies [11–14]. Recently, we showed that THG microscopy could also be used to visualize the LCN without staining in ex vivo bone samples, thereby providing a label-free alternative to confocal and 2PEF imaging [15]. These observations were also confirmed in vivo on small volumes of mouse cranial bone [16].

In the present study, we investigate the LCN of a mouse model subjected to microgravity conditions, which has previously been shown to impact the osteocyte activity and lacunar volume [17] but for which the impact on the canaliculi and network connectivity is unknown. THG imaging was preferred to confocal fluorescence microscopy since the staining was not homogeneous within the different groups, either due to the sample preparation protocol or because a fraction of the porosity was naturally occluded. This is a typical case study for the use of THG imaging of bone when fluorescence measurements cannot be performed. We therefore introduce well-suited image processing methods based on morphological filtering to analyze the THG images of the network. We then provide an analysis of the error in the structural indicators used to describe the network associated with each processing pipeline based on experimental data. These indicators allowed assessing the potential quantitative differences in the LCN of mice depending on their exposure to microgravity. Within the limits of our experimental procedure and analysis method, we show that no significant alteration of the LCN could be detected. However, important fluctuations of the LCN structure were also measured, which significance is further discussed.

## Materials and methods

### Bone samples

The samples used in this study were cut from the same bones used in Ref. [17]. Three groups of C57BL/6N male mice were considered for this study: the flight group (F, n = 4) was exposed to one month spaceflight onboard the Russian Bion-M1 satellite [18]; ground habitat control mice (synchro, S, n = 5) were placed in the same module, climate and food conditions but stayed on ground earth; ground control mice (C, n = 5) were placed in standard housing conditions. The femurs investigated were dissected and soft tissue removed immediately following euthanasia (12-24 hours after landing for the flight group), dehydrated and kept in ethanol 70% at 4 °C. The experimental design was approved by IACUC of MSU Institute of Mitoengineering (Protocol # 35, 1 November, 2012) and of Biomedical Ethics Commission of IBMP (protocol # 319, 4^th^ April, 2013). It was conducted in compliance with the European Convention for the Protection of Vertebrate Animals used for Experimental and Other Scientific Purposes.

Transverse sections of 300–500 *μm* in thickness were cut using a diamond wire saw (Well, Escil, Chassieu, France) between the proximal epiphysis and the mid-diaphysis, close to the trochanter region. To improve the quality of the transmission microscopy observations, the samples were polished to decrease the surface roughness and were reduced to a similar thickness to provide comparable conditions for all samples of the different groups. For this purpose, the samples were dried in air for 24 h, then placed at the bottom of cylindrical plastic molds of 9.4 mm in diameter and 5 mm in height and embedded in epoxy resin (Epofix, Struers) to facilitate manipulation. The embedded samples were fixed on a polishing block using double sided tape and reduced in thickness by lapping (Minitech 233, Presi) using 2000 grade SiC paper until the full cortical outermost surface (in cross section) was apparent. The samples were then unmounted, fixed with their apparent surface facing the base of the sample holder and further reduced in thickness until the other surface of the sample became apparent and the target thickness of 200 *μm* was reached using a stainless steel shim. This process ensured a good surface quality on both sides and a high degree of parallelism between the surfaces. Because of its high viscosity, the resin generally doesn’t penetrate the LCN more than 50 *μm* at maximum. One goal of the polishing procedure is therefore to remove this occluded layer and allow exposing the LCN porosity at the sample surface to improve staining using 0.02% Rhodamine B in glycerol for 3 days. However, in this study, several samples could not be stained satisfactorily. The samples were therefore mounted in unstained glycerol for THG imaging. Additionally, one sample of each group for which the staining was judged satisfactory was imaged using confocal fluorescence microscopy for validation.

### THG imaging

THG images were acquired using a custom-built laser scanning microscope (Fig 1b) equipped with a femtosecond infrared source (Insight DS+, Newport SpectraPhysics, USA), galvanometer mirrors (GSI Lumonics, USA) and a water-immersion objective (UPLSApo 60xW, 1.2NA, Olympus, Japan). The excitation wavelength was 1.18 *μm* with a pulse duration of 100 fs at sample position and the excitation power was adjusted as a function of depth in the range 40–150 mW using a motorized wave plate and a polarizing beamsplitter cube. The pixel dwell time was 5 *μs*. The THG signals were detected in the forward direction through a bandpass filter (FF01-377/50–25, Semrock, USA) using photomultiplier modules (SensTech, UK) and CPLD-based counting electronics (LCMX0 2280, Lattice Semiconductor, Portland, OR, USA) working at up to 100 MHz. The scanning and acquisition were synchronized using a lab-written LabVIEW software and a multichannel I/O board (PCI-6115, National Instruments, USA). Experimental resolutions were found to be around 430 nm (lateral) and 1.8 *μm* (axial).

For each group, a mosaic THG image was acquired for one sample by scanning the whole cortical shell of the transverse section (XY plane) in contiguous regions of 272 × 272 *μm*^2^ with a sampling of 200 × 200 *nm*^2^. In addition, for all samples, a series of images was acquired in the axial direction (Z-stack) over a single field of view in XY, by displacing the sample in 300 nm steps up to 30 *μm* in depth for each anatomical quadrant: anterior (A), posterior (P), medial (M), and lateral (L).

### Confocal imaging

A stained sample of each group was imaged using confocal fluorescence microscopy to compare with THG images. The images were acquired using a motorized inverted confocal microscope (SP8, Leica). Rhodamine–B was excited at 561 nm and the fluorescence was collected in the 566–666 nm range on a hybrid detector. The excitation power at the focal point was automatically adjusted as a function of depth in the range 6–70 *μW*. The pixel dwell time was 1.8 *μs*. The 40× (1.3NA) oil objective used provided a theoretical resolution (in the absence of aberrations) of 230 nm (lateral) and 1.0 *μm* (axial). Experimental resolutions were found to be around 340 nm (lateral) and 1.3 *μm* (axial).

### Image processing and analysis

The analysis of images detailed in section 3 was performed using ImageJ (vesselness filtering, skeletonization, quantification) using available plugins. The vesselness filtering was performed using the “Frangi’s vesselness” plugin [19] in two dimensions. A single scale was defined since the canaliculi were found to be relatively homogeneous in diameter at our resolution level. This filter essentially consists in applying a gaussian smoothing and calculating the diagonalized Hessian Matrix (i.e. the second derivatives) of the image. Frangi et al. [19] showed that several ratios can be calculated based on the sorted eigenvalues of the Hessian matrix to enhance regions of specific morphologies: plates, blobs or, as in our case, tubes. To best preserve the canaliculi, the smoothing was performed using full-width at half-maximum (FWHM) of 2.35 pixels (i.e. a standard deviation of 1 pixel). This relatively low value allowed best preserving the canaliculi edges, the downside being that a non-negigeable amount of noise was retained. Therefore, the segmentation of the resulting images was performed using a hysteresis threshold in which two thresholds were defined based on visual examination: the values above the highest threshold were set to 1, those below the lower threshold were set to 0 and those in between the two thresholds were set according to their neighboring values. The skeletonization process was then performed using the Skeletonize plugin [20]. The skeletonized data were analyzed using the AnalyzeSkeleton plugin [21] which directly provides the number of connections and positions of each canaliculi. To obtain a more global description of the relative in-plane orientation of the canaliculi, the 2D module of the fast Fourier transform (2D FFT) of THG images was calculated after vesselness filtering using custom scripts written in Python.

## Results

### Processing of THG images and extraction of relevant parameters for quantitative analysis of the LCN

**Fig 2:**
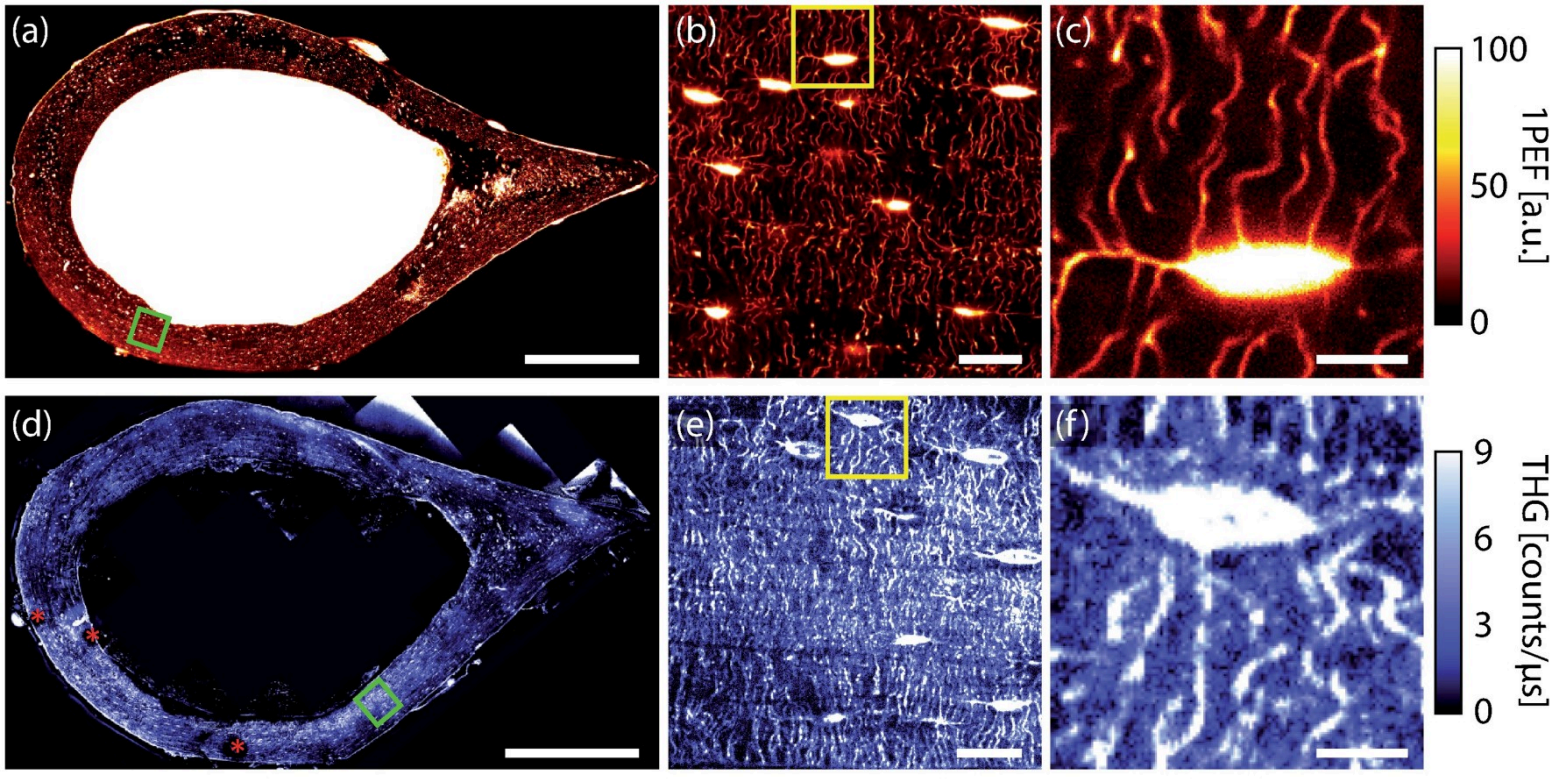
Comparison between confocal fluorescence microscopy (a,b,c) and THG imaging (d,e,f) of two different samples. (a,d) 2D composite images of full transverse cross-sections of a mouse femur. Regions shown in (b,e) are indicated by a green box in (a,d). Regions in (c,f) are indicated by a yellow box in (b,e). Red stars in (d) indicate the presence of air bubbles due to localized heating by absorption of the excitation light in the presence of polishing residues. Scale bar 500 *μm* (a,d), 20 *μm* (b,e) and 5 *μm* (c,f).

A comparison between a stained sample imaged by confocal fluorescence microscopy and an unstained one visualized by THG imaging is shown in Fig 2. The lacunae hosting the cell bodies of the osteocytes appear as bright ellipsoids in fluorescence microscopy (Fig 2c) and the canaliculi surrounding the cellular processes that connect neighboring cells are clearly visible. Similarly, lacunae and canaliculi can be observed in the THG images (Fig 2f) throughout the whole sample. One practical limitation experienced with THG imaging is the formation of bubbles during scanning (stars in Fig 2d), due to the presence of polishing residues that strongly absorb at the selected excitation wavelength, giving rise to localized heating. Nevertheless, in all THG images, the LCN was clearly visible.

The coherent nature of the THG signal results in a complex signal buildup. In biological tissues, THG is intrinsically mostly forward directed although in thick, scattering, weakly absorbing samples, a significant part of the signal can be redirected backwards [22]. For the thin slices used in this study, we therefore only detected the forward-directed THG signal which was 100 times stronger than this collected in epidetection. In the case of bone, in addition to the signal generated by the LCN itself, the mineralized collagen matrix also produces a significant, fluctuating background signal (see e.g. Fig 2f). This greatly reduces the signal-to-background ratio of the structures of interest of the LCN in THG images, irrespective of the high signal-to-noise ratio. Because both effects are intrinsic to the nature of the material, the contrast in the THG images is reduced compared to fluorescence confocal images. This effect was characterized in details in a previous study, in which a quantitative comparison of the signal-to-background ratios in confocal and THG microscopy of the LCN for various sample preparation modes was provided [15]. Extracting reliable quantitative parameters from the THG images is thus more challenging than with confocal images and the resulting error on the retrieved network is significantly increased when using similar image processing pipelines.

**Fig 3:**
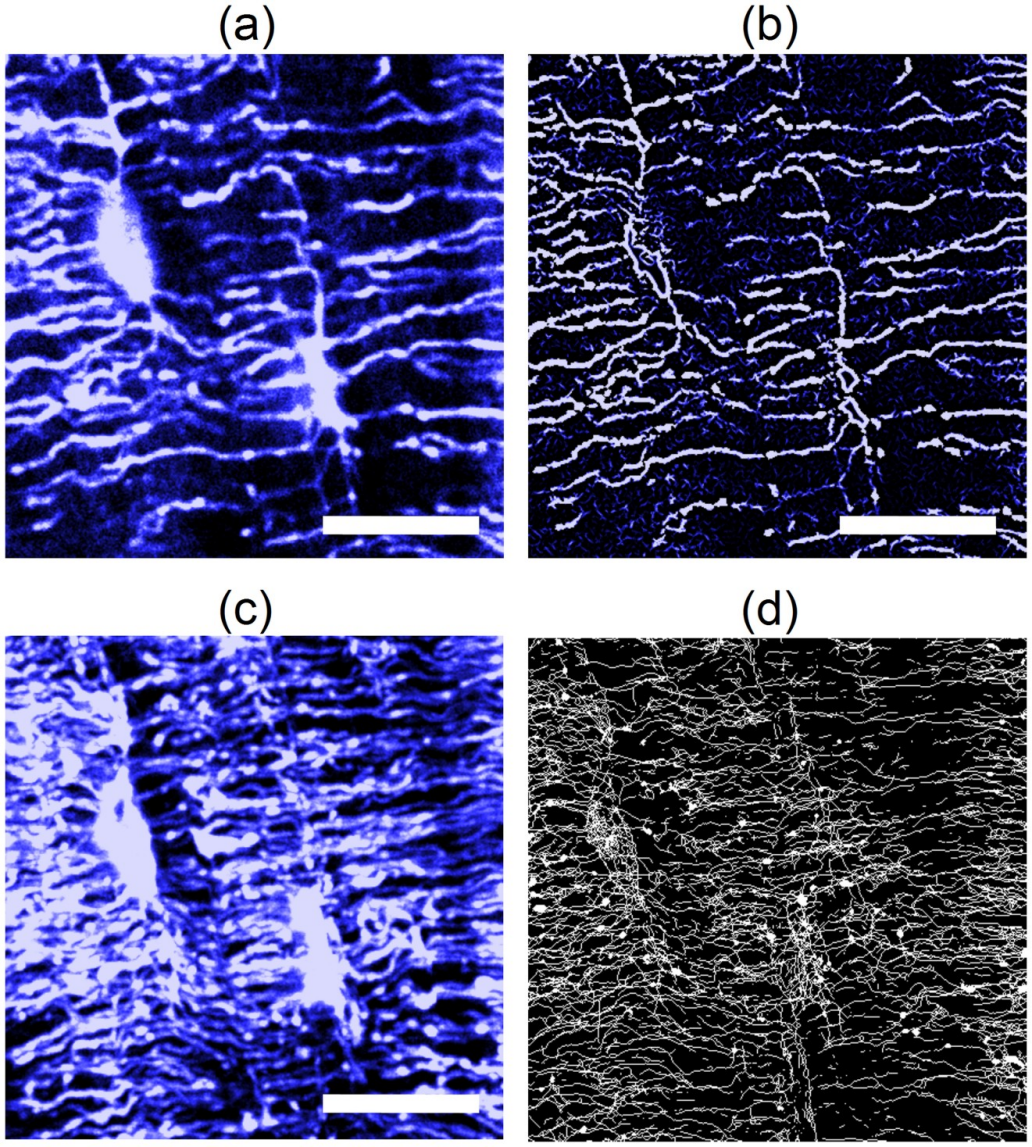
Image processing pipeline. (a) Zoom of a region of interest of a THG image at 3.6 *μm* from the surface. (b) Corresponding image after vesselness filtering. (c) Intensity projection of the full image stack along Z. (d) Maximum intensity projection of the result of the skeletonization of the filtered data. Scale bar 20 *μm*.

To analyze the THG images, we used a morphological Vesselness filter [19] which proved efficient to enhance the contrast of tubular structures before segmentation of the 3D fluorescence confocal volumes. A 2D filter was applied on all successive THG images of a stack of images prior to segmentation of the whole volume. The accuracy of our quantification method was tested on a representative volume of 64 × 64 × 12 *μm*^3^ (Fig 3) by comparing the value of two representative parameters of the network (canalicular density and connection density) with those obtained on a manually corrected vesselness-filtered skeleton based on visual inspection of the THG image of the same volume. This ensures that the reference skeleton provides an accurate description of the acquired data: artifacts resulting from noise, which did not appear as canaliculi (isolated group of pixels) on the raw THG images were removed and missing connections or incomplete segments were added following the shortest path (typically 4 pixels). The skeleton of the LCN obtained on the same test region from vesselness-filtered images was then compared to this reference dataset.

The vesselness filter proved to be effective in enhancing the faintest signals from the canaliculi. However, one drawback of this procedure is that the lacunae filtered by vesselness give rise to an artificial number of segments and connections which are best observed in the maximum intensity projection of the skeletonized image stack along the Z direction (Fig 3d). We thus evaluated the resulting error by manually creating a mask of the lacunae present in the test volume by thresholding the original image followed by erosion, to remove the canaliculi, and dilation, to restore the lacunar volume. Additional artifacts were manually removed. Applying this mask allowed removing the lacunae volume before performing the error analysis and, thus, estimating the error fraction due to the sole lacunae.

A summary of the results is shown in Table 1. In this study, we mainly focus on the total percentage of canalicular porosity, which can be compared to the existing literature. This parameter, designated as Ca.V/TV in Ref. [23], can be estimated from the total measured length of the skeleton assuming a circular cross-section of the canaliculi with an average diameter of 200 nm, which is an upper bound for mice [24]. The number and density of connections between segments of canaliculi, also designated as nodes in Ref. [25] or hubs in Ref. [26], describe the degree of branching, an additional essential information on the network structure. The lacunae were analyzed independently by directly thresholding the raw THG images in order to provide the average number of lacunae per unit volume, so-called N.Lc/TV.

**Table 1:** Uncertainty estimation for the proposed image processing methods. Two parameters are used to characterize the canalicular network: the density of canalicular porosity, and the volumetric density of connections. Skeletons obtained with different processing methods are compared with a reference skeleton that was manually corrected to match the network visible on the original THG image. In each case, the full volume is used, or a part corresponding to the position of the lacuna is removed from the analysis. % uncertainty indicated in parenthesis.

	Canaliculi		Connections	
	Number	Density (%)	Number	Density (%)
Corrected manually	1777	1.63	832	0.094
Vesselness	2020 (13%)	1.94 (19%)	1000 (20%)	0.113 (20%)
Vesselness + lacuna mask	1967 (11%)	1.88 (15%)	965 (16%)	0.109 (16%)

It should be noted, here, that such comparison allows estimating the performance of the data processing pipeline, but not that of the imaging process. Such validation has already been proposed experimentally by comparing THG and confocal images on an identical region of a test sample [15].

### Influence of microgravity on the LCN structure

Using the image processing method described above, we now study the influence of a stay in microgravity on the structure of the LCN. While changes in osteocyte activity and lacunar volume were recently demonstrated by Gerbaix et al. [17], little is known about the influence of microgravity on the LCN remodeling. We thus assess here the morphological changes of the network based both on visual inspection of the THG images and on quantification of some of the network parameters. In this study, we did not focus on the morphological characteristics of lacunae (in particular volume changes) as a proper assessment of these parameters would require a very detailed understanding of the THG signal buildup at the lacunae boundaries, and how it is possibly influenced by the addition of fresh bone matrix.

#### Morphological characteristics of the LCN

**Fig 4:**
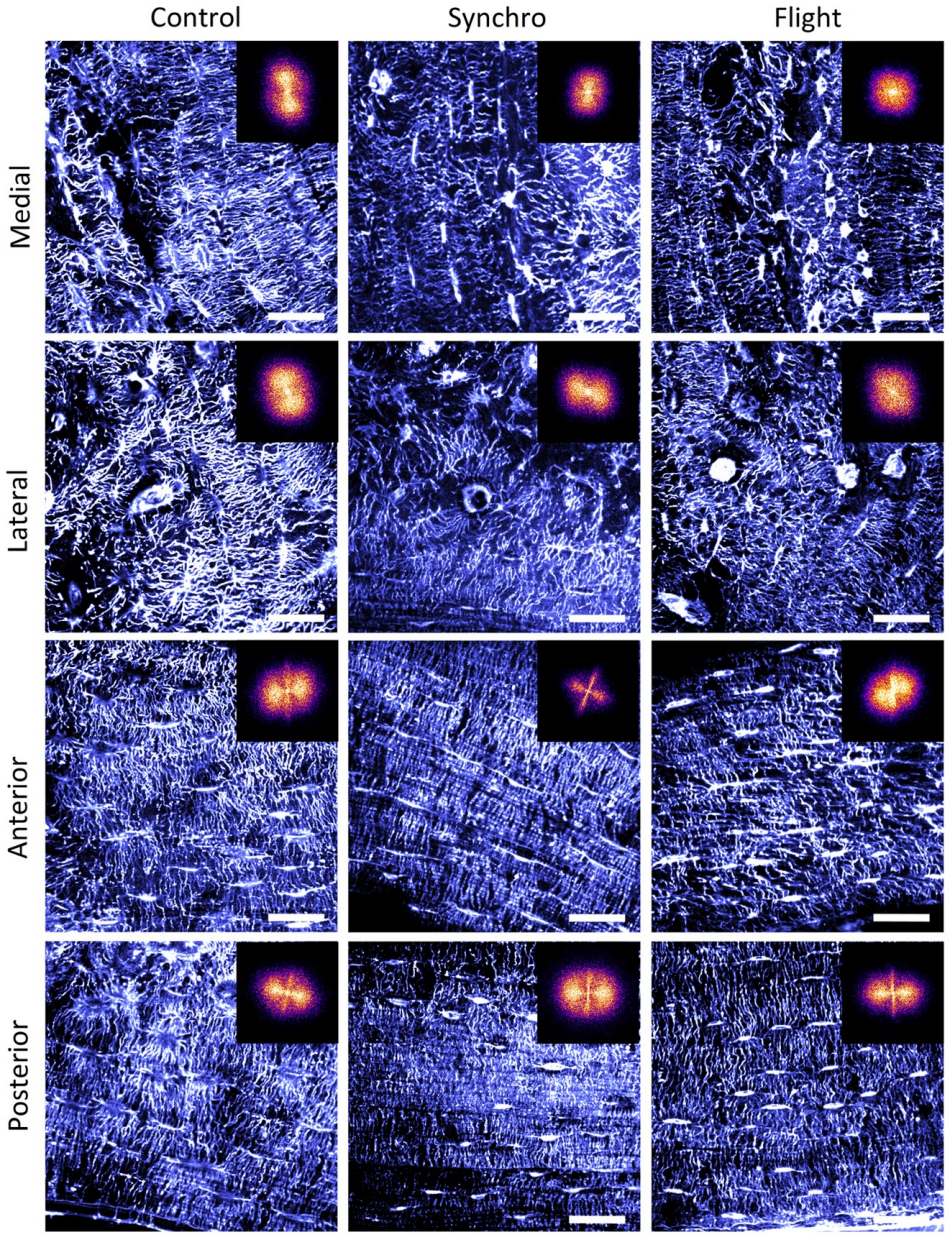
Anatomical variability of the LCN. THG images of the LCN of a sample from the control (left), synchro (center) and flight (right) groups in the M, L, A and P regions (from top to bottom). The inset shows the module of the Fourier-transformed images after vesseleness filtering.Scale bar 30 *μm*.

Significant variability in the LCN organization was observed for all samples, as illustrated in Fig 4, suggesting strong intra-individual fluctuations. However, qualitatively, two anatomical groups exhibiting similar morphological characteristics were identified: in the anterior and posterior (A,P) regions, the lacunae appear to be elongated along the tangential direction (i.e. along the bone radius), while in the medial and lateral (M,L) parts, the lacunae were rounder and the direction of the canaliculi more random, as previously reported by other authors [25]. Altogether, the degree of ordering seems stronger in the A,P than in the M,L regions. In particular, the LCN in the lateral quadrant is much more erratic, which seems to be correlated with the proximity to the trochanter, a region of muscle attachment.

This difference in canaliculi organization is reflected by the corresponding 2D FFT images shown as insets in Fig 4. Three distinct morphologies can be observed: a circular shape, as for the lateral sections of all groups, or the medial quadrant of the flight sample; an extended signal with two lobes at 180 ° from one to another, as for the medial quadrant of the control and synchro; and finally a butterfly shape which, in addition to the extended signal, exhibits a sharper streak at ∼ 90 ° with respect to the lobes, as observed in all anterior and posterior regions of all groups. This reflects the observation of a bimodal distribution of orientations, with canaliculi oriented perpendicular to the elongated lacunae. These orientations correspond, respectively, to the transverse and tangential directions of the cortical section (approximately vertical and horizontal directions in Fig 4).

Compared to these intra-individual variations, differences between flight, synchro and control groups are, at most, of low amplitude, suggesting that morphological changes such as orientation at intermediate and large scales due to microgravity are small in our experimental conditions compared to intra- and inter-individual variations.

### Quantitative assessment of network changes under microgravity

**Fig 5:**
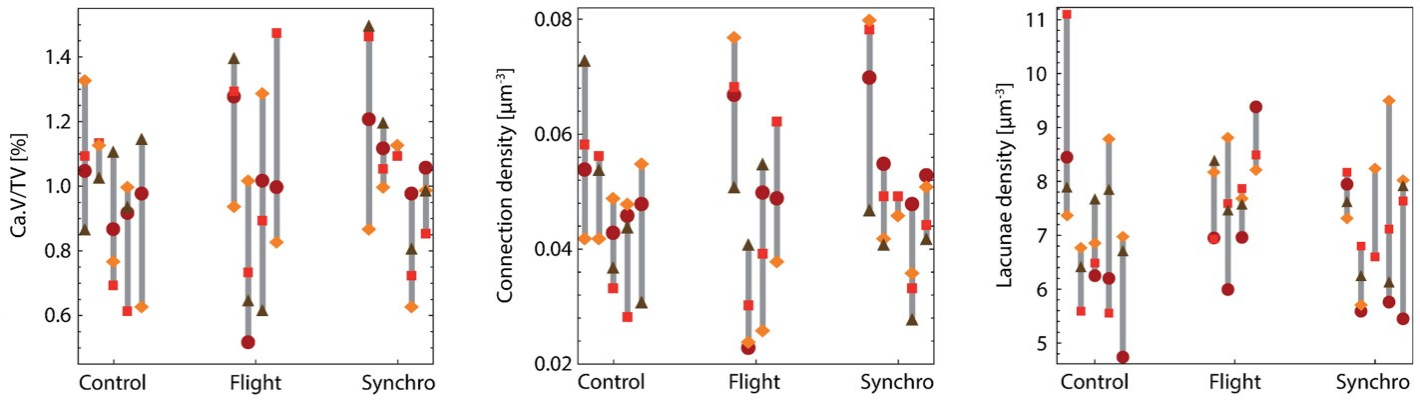
LCN parameters. Bone volume fraction of canalicular porosity (Ca.V/TV), density of connections and density of lacunae for Control, Flight and Synchro groups, for all four quadrants (light squares: lateral;dark squares: medial;diamonds: anterior/posterior). Intra-individual inter-individual variations within one group are large compared to measurement uncertainties and to the differences between groups.

To complement the above qualitative observations, we performed a more quantitative assessment of the network characteristics, focusing on its density. The canalicular volume fraction, Ca.V/TV, the number of lacunae per unit volume, N.Lc/TV, and the number of connections per unit volume, N.Connect/TV, were computed from skeletonized images (Table 2 and Fig 5). In all groups, the average volume fraction of canaliculi lies between 0.5 and 1.5%, which is in good agreement with the typical values of 0.7% found by other authors for healthy mice cortical bone [3]. Similarly, the number of lacunae per unit volume (number density) lies in the range of 6.10^4^–8.10^4^ *mm*^−3^ which fits well with the literature [27, 28].

**Table 2:** LCN characteristics.

		Mean ±SD			*p*–values (ANOVA)		
		Control	Synchro	Flight	Control/synchro	Synchro/flight	Global
N.Lc/BV (x10^4^.mm^−3^)	Lateral	6.41 ±1.52	6.18 ±1.19	7.29 ±1.45	0.82	0.28	0.52
	Medial	7.16 ±2.65	7.26 ±0.64	7.71 ±0.67	0.94	0.34	0.87
	Anterior/Posterior	7.32 ±0.72	7.42 ±1.30	8.01 ±0.50	0.86	0.22	0.24
	All regions	7.32 ±0.72	7.42 ±1.30	8.01 ±0.50	0.15	0.08	0.20
Ca.V/TV (%)	Lateral	0.96 ±0.08	1.09 ±0.10	0.96 ±0.32	0.07	0.44	0.54
	Medial	0.88 ±0.27	1.03 ±0.28	1.09 ±0.35	0.43	0.78	0.59
	Anterior/Posterior	1.00 ±0.20	1.00 ±0.18	0.95 ±0.30	0.87	0.73	0.92
	All regions	1.00 ±0.20	1.00 ±0.18	0.95 ±0.30	0.29	0.66	0.64
N.Connect/TV (x10^7^.mm^−3^)	Lateral	4.8 ±0.5	5.6 ±0.9	4.7 ±1.8	0.15	0.94	0.81
	Medial	4.4 ±1.5	5.0 ±1.7	5.0 ±1.8	0.54	0.94	0.81
	Anterior/Posterior	4.7 ±1.1	4.6 ±1.4	4.5 ±1.8	0.79	0.87	0.92
	All regions	4.7 ±1.1	4.6 ±1.4	4.5 ±1.8	0.50	0.60	0.79

Focusing on the differences between groups, we compared the values obtained for different quadrants to test for significant differences using a one-way ANOVA analysis. Statistically, no significant differences were observed between the different groups for the canalicular volume fraction, the lacunae density or the connection density. This result was not modified when pooling all regions together. Indeed, a graphical representation of the data (Fig 5) emphasizes the large dispersion of values for different quadrants in one individual, or for one given quadrant between different mice of the same group (typically +/- 50% in all groups). This dispersion is much larger than the quantification errors (Table 1), which a posteriori validates our processing pipeline in the context of this study. It is also much larger than the observed differences between groups: our data hence indicate that the extent of the remodeling of the porosity network after a one-month stay in microgravity is small compared to the intra- and inter-individual variations. In addition, no statistical differences were found between the different quadrants (lateral, medial, anterior/posterior) in either average value or dispersion.

From our set of data, we can further analyze the data dispersion to estimate the number of individuals per group that, given the intrinsic variations observed in each group, would be required to detect a significant variation in any of the parameters chosen to characterize the network. The results are shown in Table 3: first, experimentally computed mean and standard deviations were used to estimate the true mean and standard deviation of each group. The size of synchro and control groups was then computed such that the difference in mean between the synchro and flight group became significant, using p-values of 0.05 and 0.10. Similarly, the average of the standard deviations of the two groups was used to compute the group size required to detect a 10% difference between the two groups, for p-values of 0.05 and 0.10. In both cases, group sizes larger than 25 mice would be required to detect a significant change in the LCN properties due to the large intra- and inter-individual variations.

**Table 3:** Theoretical group size required to measure porosity network variations in the present study. Averages over the four quadrants were used for this calculation but little variations are observed when considering one given anatomical region.

	Mean ±SD		Group size			
			Using experimental values		10% in average values	
	Synchro	Flight	*p* = 0.10	*p* = 0.05	*p* = 0.10	*p* = 0.05
N.Lc/BV (x10^4^.mm^−3^)	7.42 ±1.30	8.01 ±0.50	33	39	25	30
Ca.V/TV (%)	1.00 ±0.18	0.95 ±0.30	49	58	26	31
N.Connect/TV (x10^7^.mm^−3^)	4.60 ±1.40	4.50 ±1.80	114	136	27	32

## Discussion and conclusion

In this paper, we demonstrate the use of THG microscopy to image and quantify the properties of the lacuno-canalicular network on large scales in the context of a biomedical study. Here, THG imaging was applied to thin, resin-embedded sections of murine femoral bones. While confocal fluorescence microscopy is routinely used to characterize optically the LCN in bone, this method fully relies on the infiltration of the cellular porosity by a fluorescent dye. In the present case, our staining protocol, which proved highly efficient for a broad variety of bones (including mouse) [15], did not provide sufficient fluorescence signal to image all the samples using this method. Due to the limited amount of material for this study, our hypothesis is that the embedding resin may have at least partly obstructed the cavities throughout the whole section, hence preventing any liquid from penetrating the LCN. The use of THG microscopy thus offers an interesting alternative to other methods such as X-ray microtomography which requires synchrotron radiation to achieve sufficient spatial resolution, and is shown here to provide suitable data for qualitative and quantitative analysis in a biomedical context.

Due to its intrinsic contrast that is not enhanced through the use of specific markers, THG imaging of the LCN requires, nevertheless, a specific processing pipeline to accurately reconstruct the network in the presence of a significant and spatially-varying background. Here, we show that the use of a well-chosen morphological filter permits a satisfying quantification of the parameters characterizing the network with minimal complexity and computation time. We quantified the corresponding errors and found them to be in the range 10–20%: this figure could be further reduced by increasing the complexity of the processing algorithms (including e.g. a deconvolution step) or of the image acquisition process (by use for example of adaptive optics to improve image quality at intermediate depths [29]). In this study, however, we found that the quantification errors were small compared to sample-to-sample variations.

Focusing on the biomedical question that motivated this methodological development, our analysis did not point to any significant changes in the LCN characteristics of mice subjected to microgravity, nor in the synchro group, at least based on the three chosen parameters: the canaliculi volume fraction, the number of lacunae per unit volume and the density of connections. This is somewhat contradictory with previous results reported on rat [30], although this is a different model. Furthermore, a larger scale study of those samples showed that, while the structural properties of the mineralized tissue were not significantly altered in the flight group, a high level of osteocyte apoptosis was observed in the femoral mid-diaphysis which was correlated with a decreased lacunar volume [17]. However, it should be noted that this study focuses on the porosity network rather than on the cellular network itself: while our data suggests that the LCN is little modified by microgravity during the time scale probed here (∼ 1 month), it is possible that this does not reflect changes in the cell network. In particular, in the case of osteocyte apoptosis and subsequent processes retraction, it is possible that the corresponding lacunae and canaliculi remain for a short period following cell death. It would therefore be informative to complement these measurements with a visualization of the cell network, which was unfortunately not feasible within the framework of this microgravity project.

It is nevertheless interesting to further analyze our results in terms of intra-group variability. The lack of significant differences in the LCN structure between the three groups could indeed partly derive from the large variability observed in each group between different animals and even more between different anatomical regions of a single animal. This is illustrated by the large group sizes that would be required to measure a significant effect of a given external stimulus (Table 3). Irrespective of the nature of this stimulus, group sizes of at least 25–30 animals would be required to detect ∼ 10% variation in the structure of the LCN: such figures thus provide a useful reference for future studies, as an order of magnitude for the dispersion in LCN characteristics are lacking so far in the literature. They also point towards the difficulty of measuring moderate variations of the network structure in the presence of such a large sample-to-sample variability. Because of the practical and ethical difficulties of performing such microgravity studies on such large sample groups, further studies of the fluctuations of the LCN would be of high added value to better target comparable regions of interest, i.e. to refine the sample preparation protocol to reduce the intra-group variations.

Also of importance, we find that such variability already exists within one single bone when considering different anatomical regions (Fig 5): this suggests that strong fluctuations may also be encountered between different locations along the femur (axial direction), and that slight variations in the position of the cut of each sample along the axial direction of the bone might increase the observed variability. Here this effect is unlikely to account for the observed fluctuations, as we do not find correlated variations for the different quadrants from one animal to the next (Fig 5). Nevertheless, the systematic study of the LCN structure at the scale of an entire bone and the estimation of the LCN structure variations at the scale of the entire organ would be of critical interest to put such comparative data in perspective.

In conclusion, we have shown here that THG imaging offers a true potential to analyze the effect on the LCN of major bone diseases, such as osteoporosis, or the effect of drugs on bone development, and we provide estimates for the intra- and inter-animal variability of the LCN structure for the design of further biomedical studies.
